# Control of a Supernumerary Robotic Hand by Foot: An Experimental Study in Virtual Reality

**DOI:** 10.1371/journal.pone.0134501

**Published:** 2015-07-30

**Authors:** Elahe Abdi, Etienne Burdet, Mohamed Bouri, Hannes Bleuler

**Affiliations:** 1 Robotic Systems Laboratory, École Polytechnique Fédérale de Lausanne (EPFL), Lausanne, Switzerland; 2 Department of Bioengineering, Imperial College of Science, Technology and Medicine, London, United Kingdom; University of Udine, ITALY

## Abstract

In the operational theater, the surgical team could highly benefit from a robotic supplementary hand under the surgeon’s full control. The surgeon may so become more autonomous; this may reduce communication errors with the assistants and take over difficult tasks such as holding tools without tremor. In this paper, we therefore examine the possibility to control a third robotic hand with one foot’s movements. Three experiments in virtual reality were designed to assess the feasibility of this control strategy, the learning curve of the subjects in different tasks and the coordination of foot movements with the two natural hands. Results show that the limbs are moved simultaneously, in parallel rather than serially. Participants’ performance improved within a few minutes of practice without any specific difficulty to complete the tasks. Subjective assessment by the subjects indicated that controlling a third hand by foot has been easy and required only negligible physical and mental efforts. The sense of ownership was reported to improve through the experiments. The mental burden was not directly related to the level of motion required by a task, but depended on the type of activity and practice. The most difficult task was moving two hands and foot in opposite directions. These results suggest that a combination of practice and appropriate tasks can enhance the learning process for controlling a robotic hand by foot.

## Introduction

The first laparoscopic surgery was performed in 1910 for diagnostic purposes [1]. During the following decades the initial method was improved resulting in a large number of laparoscopic surgeries. This type of surgery uses small incisions for inserting instruments inside the patient’s abdomen. This is beneficial for the patient, but results in confined instrument movements, limited 2D view of the operational site and loss of haptic feedback, making dexterous manipulation more difficult for the surgeon.

In the majority of cases, laparoscopic surgeons need one or several assistants to hold instruments, hand them the required tools and assist in performing tasks that could not be carried out with the two hands already fully involved. However, working with human assistants may cause complications. Each assistant will have a certain level of proficiency, may be unfamiliar with the surgeon and affected by tensions in the surgical room. Previous studies [2] have shown the important role played by the information flow between the surgeon and the surgical staff on team performance, and how a communication mistake may affect the patient safety. Nurok et al. [3] found out that teamwork is an important factor for safe and efficient surgery. Inadequate teamwork behavior was positively associated with increased odds of serious complications. Also there are fewer total errors when the surgical team is familiar with the operating surgeon. In laparoscopic surgery for instance, the assistant responsible for holding the endoscope should try not to disrupt the operating surgeon. Novice assistants often have difficulties in positioning the camera appropriately in 3D space, are confused with the fulcrum effect and suffer from fatigue [4]. Improving the accuracy and precision of surgical techniques was the first motivation for the development of robotic surgical systems [5]. Replacing human assistants with robots may solve the potential communication issues among the members of the surgical team.

Robotic devices have also be developed in recent years to assist workers in industrial environments, e.g. in holding an object on which to work [6], [7]. These robotic arms can assist the user in simple tasks such as holding a workpiece while the user performs an operation e.g. drilling. Such supernumerary robotic limbs may use vision and other sensors to observe the operator’s actions and act accordingly.

During the last 20 years some efforts have focused on replacing human assistants by robotic arms. In the surgical field the potential advantages of robots include precision, steady positioning of tools, cost effectiveness in long term and fatigue avoidance [8]. Our vision is that with more than two arms, surgeons could handle more instruments and perform more tasks independently, and fewer human assistants would be needed for complex interventions. Furthermore, the physical burden on the assistant would be decreased and, by reducing tiring long lasting operations, higher precision could be reached [9]. In an ideal operation, the surgeon should have direct control on all the devices that are involved in the intervention. Camera holders are the most common robotic assistants in operational rooms. These devices are either motion controlled (e.g. EndoAssist [10]), voice activated (e.g. AESOP [11]), or simply commanded by a joystick (e.g. LapMan [12]). In the EndoAssist (which was introduced in 1982 [13]), the movement of the camera is directed by the surgeon’s head movements using a head mounted infrared emitter and a sensor which detects the motion of the emitter. A study comparing the EndoAssist with a human camera holder found no significant difference in complication rates or total operative times. AESOP (which was first released in 1994 in USA) can be controlled either by voice, hand or foot actuated controllers [14]. Kavoussi et al. [15] compared the accuracy of AESOP with human assistant in holding the endoscope during urological laparoscopic surgery. They found that the robotic arm holds the camera in a steadier manner with less inadvertent movements, and could show that a robotic camera holder is more effective and accurate compared to a human assistant. According to Wagner et al. [16] AESOP and EndoAssist have the same surgical performance. The LapMan endoscope holder robot developed in 2004 is controlled by a joystick (LapStick) clipped onto a laparoscopic surgical instrument under the surgeon’s index finger [17].

Also in 2004, Kwon et al. [4] proposed a combination of the two main control methods used for the camera holding robots. Their robot, named KaLAR, may be controlled either by voice commands or by tracking the surgical instrument (which has already been marked). A more recent camera holder robot is the LER (light endoscope robot) developed in 2007 [18]. It is a voice-controlled robot which can be placed on the patient’s skin and is lighter and smaller than older similar robots. Finally, the commercialized ViKY [19] (also released in 2007 [18]) can be controlled either by voice commands or by a multidirectional footswitch. In the next version, the control method is expected to be improved based on visual servoing, using instrument tracking [19].

Therefore developing a suitable strategy for controlling an assistive robotic arm is of high interest for various fields. It is important that the operator can keep focused on the task with minimum distraction. The control paradigm should feel natural to the operator, as if the supernumerary hand would be like one’s own hand. This relates to research on embodiment reviewed e.g. in [20]. In 1998, Botvinick et al. [21] provided the first evidence for the possibility of inducing the perceptual illusion of owning a rubber hand. In case of a large overlap between the real and virtual bodies, visuo-proprioceptive cues are sufficient to create the illusion. However in case of minimal overlap between the virtual body and the real one, the visuo-tactile cues become important [22]. Also it has been shown that with appropriate multisensory correlations a virtual limb can be integrated into body perception [23]. This finding is consistent with the results of fMRI studies [24] showing that embodying a virtual limb through congruent visuo-tactile-proprioceptive stimulation engages the same multisensory areas that are activated when participants view their real hand being touched [25] or experience the classical rubber hand illusion [26].

The illusion of virtual limb ownership may also be evoked through imagination of a motor act accompanied by movements of a virtual hand [27]. Also, under the same conditions, the spontaneous movement of the virtual hand produces measureable muscle activity in the real arm. In 2011, Guterstam et al. [28] demonstrated the possibility of inducing the perceptual illusion of having a supernumerary right hand, such that the subject actually felt that he has a second right hand. Their findings suggest that the embodiment of a supernumerary limb is possible if it is aligned with the body in an anatomically similar fashion as the real limbs. Guterstam et al. have also demonstrated the possibility of developing a sense of ownership towards an invisible hand [29]. Other studies have shown that while multiple supernumerary limbs can be incorporated into *bodily image* (i.e., the sense of ownership towards the supernumerary limb), only one can be included in the *body schema* (the ability to control the supernumerary limb) [30]. To induce simultaneous sense of ownership towards two supernumerary rubber hands, these should be at the same distance from the subject’s hand [31]. In addition, it has been demonstrated that the size of the incorporated body part is not important and ownership illusion can be induced towards very small or large bodies [32].

The aim of the present study is to investigate the possibility of controlling a third hand using a control paradigm with minimum distraction for the user. The ultimate goal is for the control strategy to be used by surgeons for commanding a robotic hand simultaneously with their two real hands in order to perform surgical tasks. We propose tracking the foot movements for controlling the displacements of the third hand. Foot pedals are widely used for vitrectomy in microsurgery [33] and laparoscopic surgery, dentistry, etc. for turning on/off a device, as well as for commanding the endoscope movements in laparoscopic surgery. Carrozza et al. [34] have developed a wearable foot interface for controlling a prosthetic arm. Successful control of the grasp was demonstrated, basically consisting in on/off steps activated by a specific combination of pressure switches. To our knowledge no study on continuous tracking of foot movements for controlling a medical robotic arm is available in the literature.

Also there seems to be no previous study of simultaneous usage of hands and foot for assessing the appropriate level of difficulty of the tasks and the feasibility of the concept. We designed a set of experiments in virtual reality to investigate the feasibility of the proposed control strategy, and analyse the learning curve of the subjects. We also examine whether the subjects move the limbs serially one after the other, or in contrary control them in parallel. This is completed by a subjective assessment of the development of sense of ownership towards the third hand, the physical and mental load of using the foot for commanding a third arm.

These questions are investigated on three tasks (implemented as *games*) of varied difficulty involving three hands commanded by the natural hands and a foot. A first game studies the possibility of controlling three hands to complete a simple task namely displacing the hands to predefined targets. The second game investigates the capability of humans in moving the foot simultaneously with and independently of the two hands, similar to typical surgical situations where the third hand should complement the actions of the two intrinsic hands. The last game involves more active movements and subjects have to pay attention to multiple factors during the game. This is to study humans’ approach to multiple tasks management using three hands. While we refer to a surgical situation, the present study is to examine the general feasibility of controlling a third arm with a foot. The use of such as system in a specific surgery with a target user population and with given tools’ features should be tested in a dedicated study.

## Methods

### Experiment

Thirteen subjects (two females) with mean age of 24±3 years participated in the experiment. The experiment was approved by the BMI Ethics Committee for Human Behavioral Research at EPFL (Reference: BLEULER 2014 04 24), and each participant gave written informed consent prior to the experiment.

An experiment was developed to investigate how subjects coordinate the three virtual hands to complete a task. Two of the virtual hands move with the user’s real hands and the third is controlled by the right foot. Three tasks or games were designed to address specific questions. The difficulty level of a game could be controlled by the progression of skills and the mix of challenges [35]. The level of difficulty of each task is judged on the basis of the amount of motion of the two hands and of the right foot. Each of the three games is played twice.

### Setup

It is reported in the literature that a first sight of the fake or virtual body is essential in the illusion of ownership [22], and that the body shape is an important factor of the embodiment [22], [36], such that for example the ownership illusion cannot be induced for noncorporal objects like a piece of wood [29], [37]. These findings motivated us to use for the three hands a realistic view of a hand in virtual reality. Also we decided to reproduce the finger movements of the two real hands in the corresponding virtual hands.

In our setup, two Microsoft XBOX 360 Kinect depth cameras are used to track the movements of the limbs: one for the two real hands and the other for the feet. As each SDK can support only one camera, a network of two PCs is used to provide appropriate feedback of the three virtual hands in real-time. To detect the finger motions, we use the 3Gear Systems Company SDK that has a library of most popular hand gestures. The 3Gear can recognize a limited number of hand gestures, using a PC with the following minimum specifications: Intel Core i7 2.3GHz, 8 GB of RAM and Windows 7 64-bit or Mac OS X 10.8.2. In all of the games, the two gray hands move with the two real hands of the player while the yellow hand is controlled by the foot.

### First game

**Fig 1:**
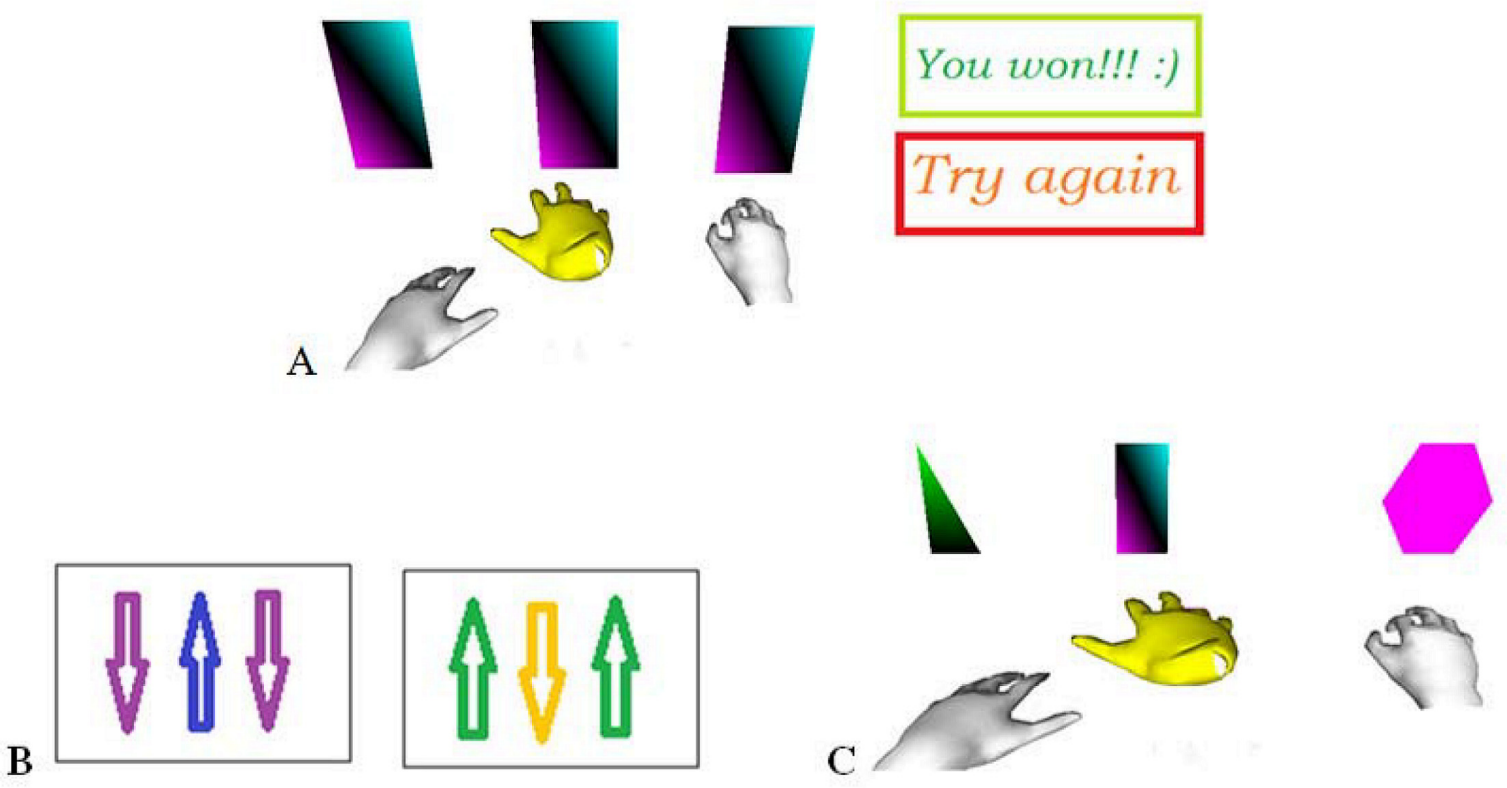
Three games to study the control of three virtual hands. A Left: Three rectangular targets and three virtual hands, Right: Automatic messages after success or failure. B: Two sets of arrows which indicate the correct direction of movement of the three virtual hands. C: Each of the three falling objects should be caught by the corresponding hand.

The first game examines the efficacy of controlling a third hand by the foot simultaneously to the two hands. Three rectangular targets appear on the screen on a horizontal line, as well as three virtual hands (Fig 1A). The left and right hands have to touch the left and right rectangular targets, respectively. The middle rectangle can be touched with the (yellow) foot-controlled hand. The “touch” is defined as a brief contact between the hand center and the object. Each target is sensitive only to its allocated hand i.e. touching an object with a wrong hand will have no effect. The objective of this game is “to touch all the three targets simultaneously and as fast as possible”. If the time elapsed between touching the first and the last object is less than four seconds, this is interpreted as a success and the message “You won!” appears on the screen. Otherwise, the message “Try again!” will show up, indicating a failure. The moment at which each object is touched is recorded as well as the success or failure in each trial.

### Second game

The second game studies the simultaneous control of the two hands in a direction opposite to the foot. Again, three rectangles appear on the screen, with a set of three arrows showing the desired direction of movement of each rectangle (Fig 1B). The arrows indicate that the rectangles on the left and right sides should move in the same direction and the middle rectangle should move in the opposite direction. Each rectangle can be moved only by the respective hand. The task consists in moving the three targets in the correct direction simultaneously for at least three seconds. Once the goal is reached, the three arrows change so that the user has to move each rectangle in the opposite direction. Two sets of arrows are used in the game, which appear twice on the screen one after another, resulting in a total of four rounds per game. For each participant, the time required to complete the game is recorded. Note that many other combinations of the hand-foot simultaneous movements could be investigated, e.g. one hand and one foot moving in the direction opposite to the other hand. The directions combination selected for this game corresponds to most surgery operational situations where the two hands collaborate in performing a task while the foot should perform independent, but complementary movements.

### Third game

The third game studies the coordination of the two hands and the foot in performing a more complicated task. Three objects fall from the top of the screen, the user has to “catch” them before they reach the bottom (Fig 1C). The left and right objects have to be caught by pinching with the corresponding (grey) hands while the middle object has to be “touched” with the foot controlled (yellow) virtual hand. When an object is caught successfully, it returns to the top and starts falling again. The speed of the objects increases after each of them has been caught at least three times. Only three levels of speed are used: Once the maximum speed is reached, it will remain constant regardless of the number of objects successfully caught. If the user fails to catch four objects, the game will be terminated with a failure message. If every object is caught three times at the maximum speed level, the game will terminate with a success message. Every subject played the three games in the above order and each game was played twice.

### Assessment

Our assessment of the subjects’ performance is based on the time required to successfully complete a task. In the third game, the number of missed objects is used as an additional measure of efficiency. The assessment is completed by a questionnaire about different aspects of the control strategy, which is filled after each game. The three games are played in the same order as described above. The questionnaire consists of grading the statements of Table 1 using a Likert scale, from 1 (for “strong disagreement”) to 5 (for “strong agreement”). Participants also answer the two comparative questions of Table 2 when the whole experiment is completed.

**Table 1:** Questionnaire statements.

	Questionnaire statements
1	It felt natural for me to control three hands simultaneously
2	It was easy for me to control the third hand by foot
3	I felt as if the virtual third hand was my own
4	Playing the game was physically tiring for me
5	Playing the game was mentally tiring for me

**Table 2:** Comparative questions for the three games.

	Questionnaire statements	First game	Second game	Third game
1	In which experiment was it easier for you to control the third hand? (Put the maximum 3 for the easiest)			
2	Which experiment helped you more in mastering the simultaneous control of three hands? (Put the maximum 3 for the most helpful)			

### Statistical analysis

Normality of the data sets was checked using the Jarque-Bera test. For normally distributed data sets a t-test was used to identify significant differences between different sets, with significance level p<5%. The nonparametric Wilcoxon signed rank test was used to analyze the differences between two non-normally distributed data sets.

## Results

**Fig 2:**
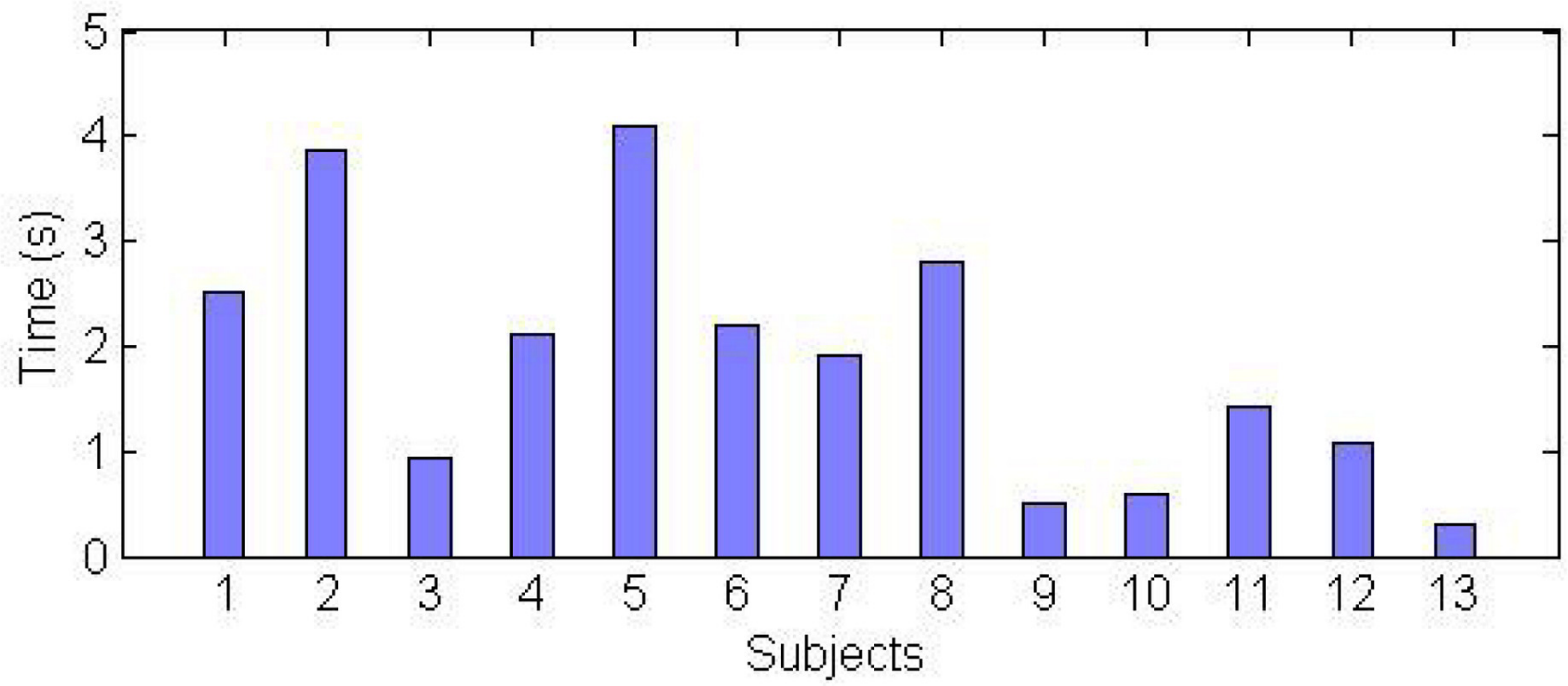
Average performance time for each participant for the first game.

The first game was successfully completed by all 13 subjects. Nine subjects succeeded in all trials and four failed once. To infer performance we consider the *coordination time* between touching the first and the last rectangles. The average coordination time over the subjects and two trials of 1.87±1.14s indicates a good coordination of the three virtual hands. However, Fig 2 exhibits a large variation between the mean coordination times of different subjects (with a 10 fold duration variation between some subjects). This suggests important individual differences in the ability to simultaneously control three limbs between the subjects.

**Fig 3:**
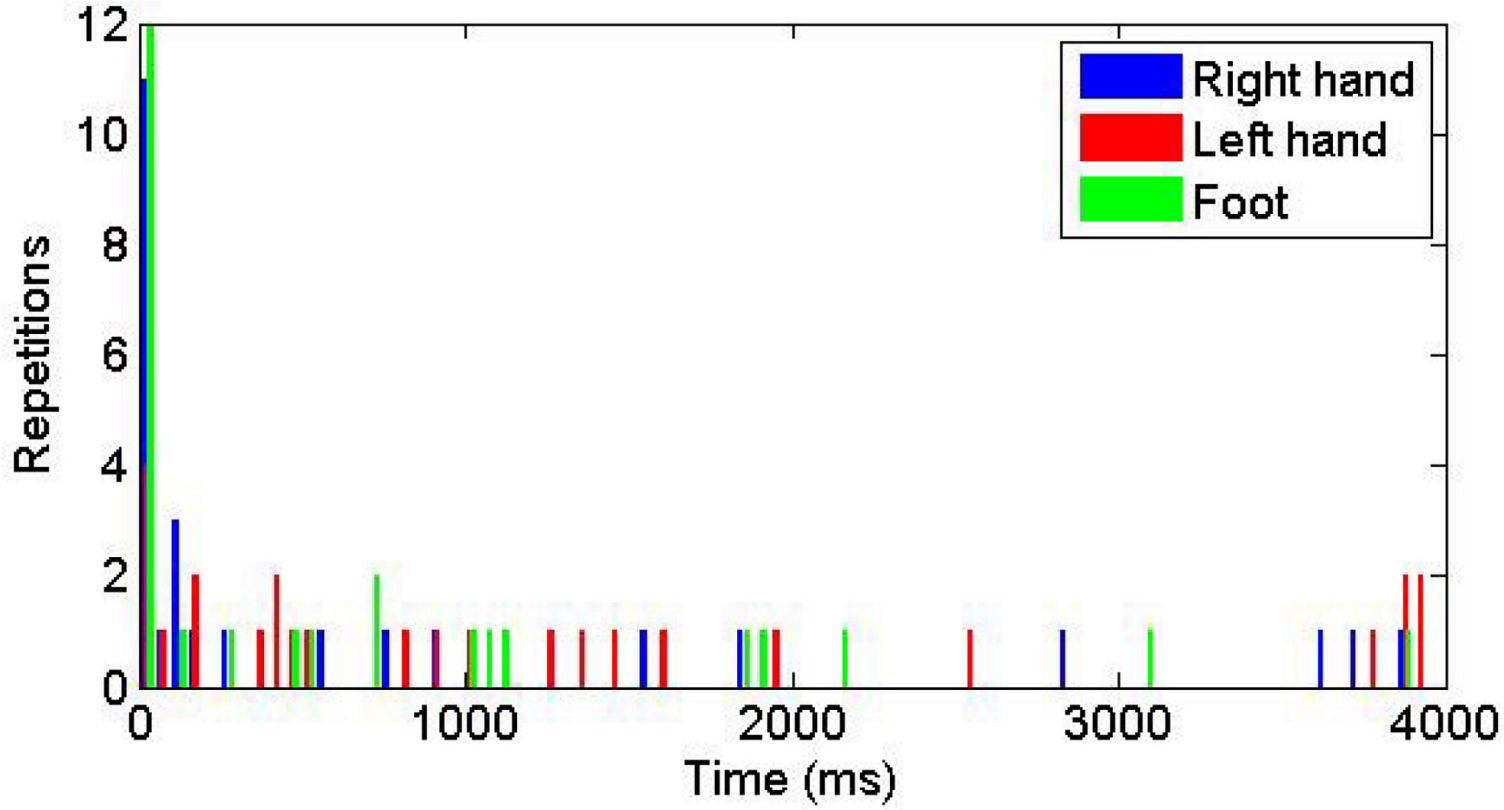
Histogram of the time to reach the three targets. 0ms corresponds to the time of reaching the first target.

Participants were free to choose the sequence of touching the rectangles i.e. a subject could start with touching the right rectangle, then continue with touching the left rectangle and the middle one or they could choose any other sequence. Fig 3 shows the distribution of times to reach the three targets. A first hypothesis is that to simplify computation of the movement, the subjects would move the virtual hands in a definite order. If the movements of the three hands would be carried out serially, we would see separated distributions of the arrival to each target. In contrast the overlap of the temporal distributions of reaching the three targets suggests that there is no priority in the sequence of limb actions, and the movements of the three limbs are carried out in parallel.

The action most frequently started with the foot movement (42% of times) and the right hand (38%) and least frequently with the left hand movement (20%). Also the right (dominant) hand was used before the left hand in 64% of times, while the probability in random selection is 50%. The average coordination time was on average 14% shorter in the second as in the first trial; however this difference was not significant.

The action most frequently started with the foot movement (42% of times) and the right hand (38%) and least frequently with the left hand movement (20%). Also the right (dominant) hand was used before the left hand in 64% of times, while the probability in random selection is 50%. The average coordination time was on average 14% shorter in the second as in the first trial; however this difference was not significant.

Another hypothesis was that people would see their real hands as one group and the third hand as a separate tool. Consequently they would touch the targets that correspond to their two real hands one after another, either after or before touching the middle target by the foot controlled hand. This was again tested by recording the moment at which each target is touched. Six different conditions are possible for the succession of the two hands and foot for touching the targets, in two of which the foot is moved after one hand and before the other. Meaning that if the limbs are moved randomly, in 2/6 = 33.3% of times the hands are not moved in a row. Results show that in reality the foot comes between the two hands in 31% of times, which is close to random.

**Fig 4:**
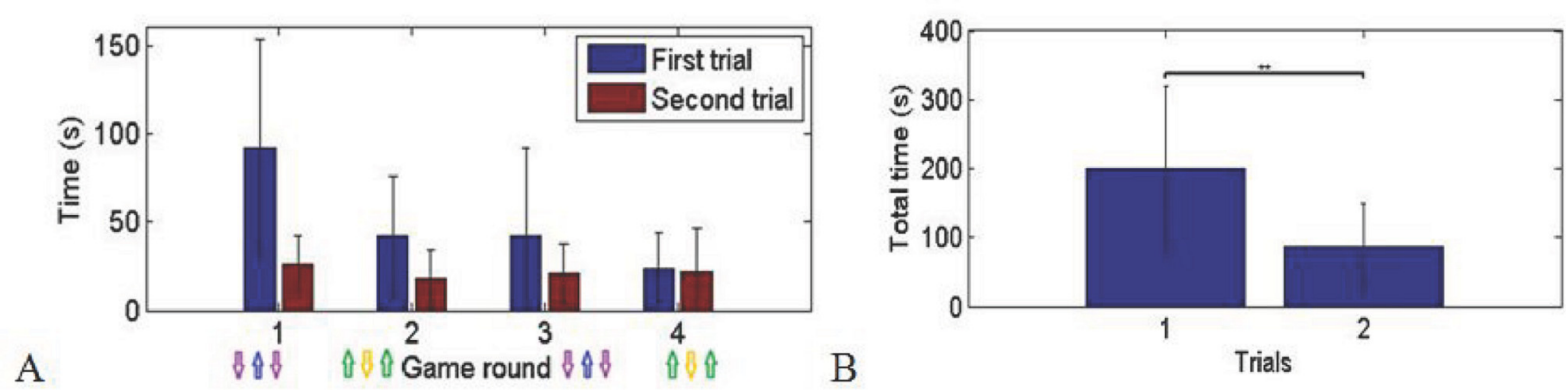
Second game: A: Average allotted time over the subjects in four rounds of two trials. B: Average total performance time in the first and the second trials (p<0.003). The standard deviations are presented in the diagrams.

In the second game participants had to move three objects, with the foot’s rectangle in opposite direction to the two hands’ objects. This corresponds to typical medical interventions where the two hands work on an operation together and the foot controlled third hand should assist. The game has four rounds, two for each of the two predefined directions. The whole game was played twice. The results show that the subjects required a significant time to keep the three hands in the correct direction (Fig 4). However the required time for completing each round decreased through the four game rounds to approximately 20 seconds. Also the allotted time in the second trial was less in all the respective game rounds. Statistical analysis shows that the performance time was longer in the first versus the second run of the game (p< 0.003, Wilcoxon signed rank test). The marked decrease in the required time for completing the game suggests that the users’ performance improved significantly within a few minutes of practice.

**Fig 5:**
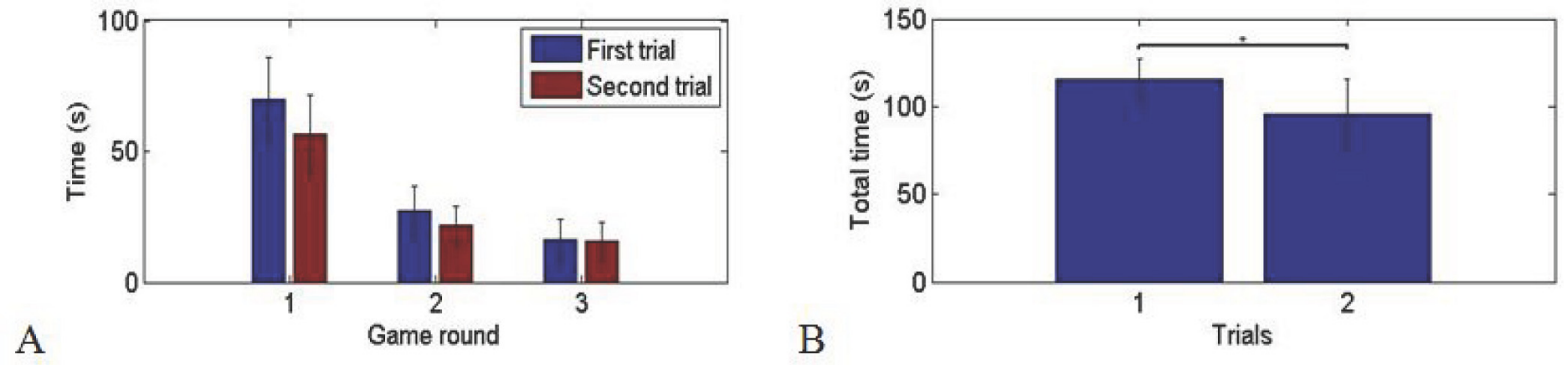
Time to complete the third game: A: Average allotted time over the subjects in three rounds of two trials. B: Average total performance time in the first and second trials (p<0.014). The standard deviations are presented in the diagrams.

The third game tested a more complicated coordination task as the first game. The results again exhibit a significant performance time decrease in the second trial as compared to the first trial (Fig 5, p<0.014, Wilcoxon signed rank test). However, here the time difference between the three rounds of the same game was also due to the increase in the falling speed of the objects corresponding to faster operation. Most of the subjects were successful in catching the falling objects. An average of 2.83 objects was lost in each trial, i.e. about 10% of the objects. The user performance again improved within few minutes of practice.

### Subjective assessment

**Fig 6:**
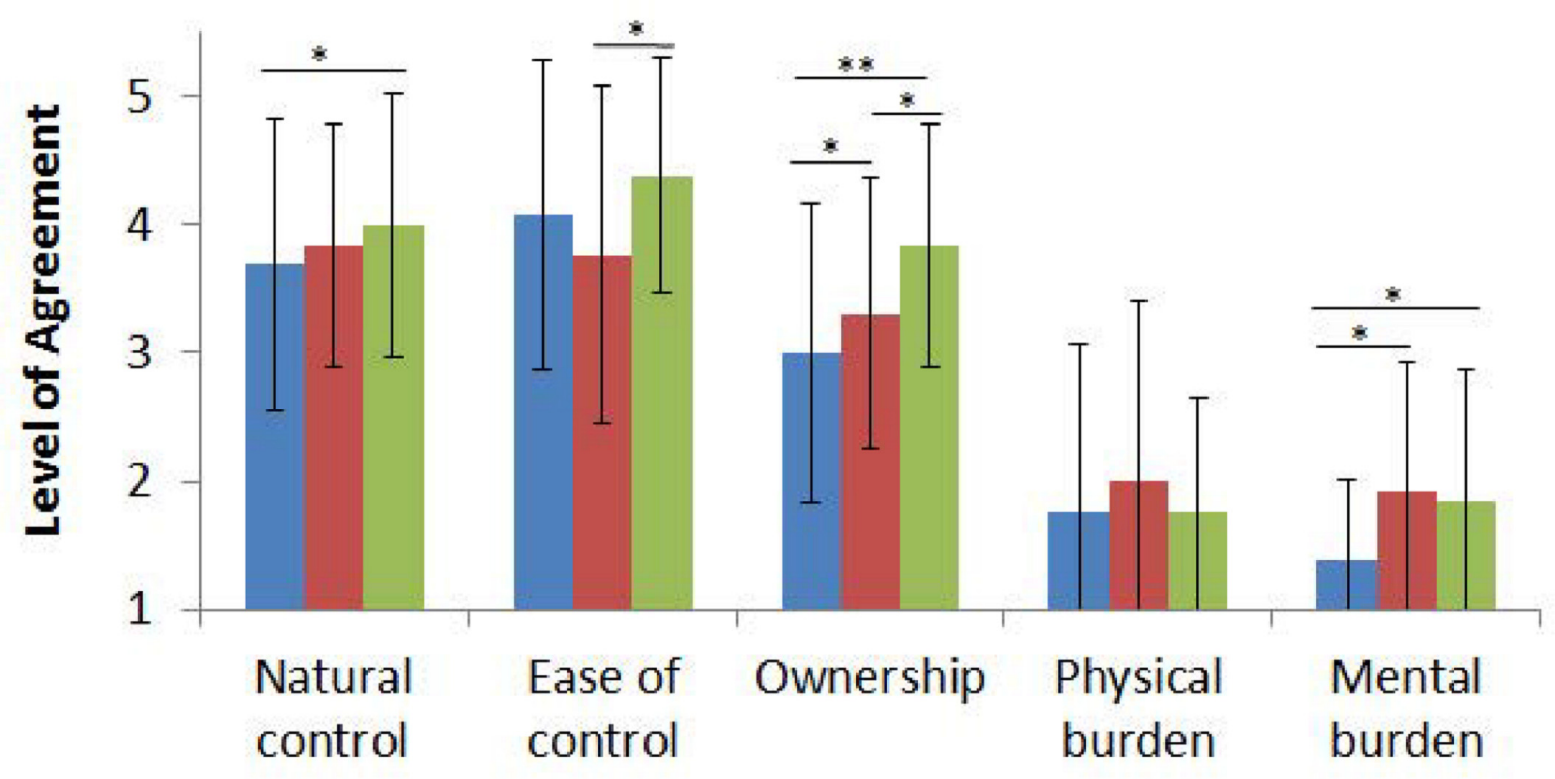
Average response of all the participants to the questionnaires for each of the three experiments (Blue, red and green respectively correspond to the first, second and third game). Statements are ranked through 1 to 5 (1: Strong disagreement, 5: Strong agreement). *: p<0.05, **: p<0.01. The standard deviations are presented in the diagram.

The results of the questionnaire about the control of the third hand are illustrated in Fig 6.

We see that the simultaneous control of three hands starts to feel more natural as the user advances from the first to the third game. In particular it feels significantly more natural in the third game compared to the first one (p< 0.040, t = -2.309, t-test). Also it has been significantly easier for the subjects to control the third hand by their foot in the third experiment compared to the second one (p<0.014, t = -2.889, t-test). This may be due to the chronological order of the experiments; as the third game comes last, users have already gained some experience during the previous two games and they feel more at ease in the third game. However, controlling the third hand was hardest in the second game, although the third game is more dynamic and in spite of the fact that users have gained some experience in the first game. This shows that apart from practice, task type may influence participants’ evaluation of the ease of an activity.

The participants generally found the physical burden low, although two subjects mentioned that it had been tiring. Mental burden was found to be low. It was lower for the first game compared to the second (p<0.016, Wilcoxon signed tank test) and the third ones (p<0.04, Wilcoxon signed rank test).

The sense of ownership does not follow exactly the reported ease of the task, i.e. the sense of ownership constantly increases from the first to the second and the third game. This improvement of the sense of ownership is significant between each two combinations of the games (first vs. second game: p<0.04, t = -2.309, t-test; second vs. third game: p<0.013, t = -2.941, t-test; first vs. third game: p<0.003, t = -3.811, t-test) indicating the strong effect of practice in developing the sense of ownership. Considering the short period of practice during the games, this suggests that ownership of the third hand can be developed to a satisfying level within a few minutes of active usage of the virtual hand.

**Fig 7:**
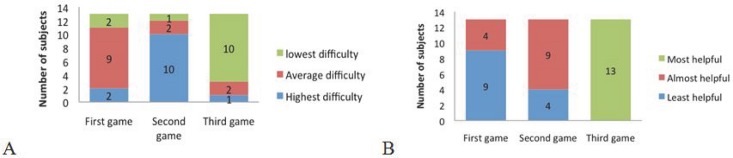
Comparative questions: A. “In which experiment it was easier for you to control the third hand?” B. “Which experiment helped you more in mastering the simultaneous control of three hands?”

At the end of the whole experiment, participants compared the three games with respect to the ease of control of the third hand. They had first to select the most difficult and the easiest game (Fig 7A). 10/13 participants stated that they have controlled the third hand more easily in the third game. Also 10 participants found the second game the most difficult one, which may indicate that the least difficulty of the third game does not stem from practice with the two first games. These results are in agreement with those of the dedicated questionnaires for each game.

All users found the third game the most helpful one in mastering the simultaneous control of three hands. They put the second and the first experience in the next levels (Fig 7B). Although the only game in which they were obliged to move all the three hands simultaneously was the second one, subjects found the third one more helpful in learning simultaneous control of three hands. This may mean that dynamic games with more interesting interfaces may be more effective in developing control skills.

## Discussion

An experimental study was conducted to evaluate the feasibility of controlling a third virtual hand by foot simultaneously with two virtual hands commanded by the real hands. Three games were designed to investigate the control of the three hands in situations abstracted from typical surgical operation conditions. The performance of the participants was assessed through monitoring their actions as well as from their answers to questionnaires filled after each experiment.

The results of the first experiment to study the simultaneous control of the three hands showed that participants used the three virtual hands without any specific priority i.e. not in a predictable sequence. This parallel control of the three hands suggests that the subjects integrated the third hand in their action as an equivalent of their two real hands.

The other games investigated performance time when i) the foot-controlled virtual hand was carrying out a different but complementary task to the two hands (second game) and ii) the three hands worked simultaneously on a dynamic task (third games). In both games the performance time decreased significantly within a few minutes of practice, which indicates a rapid learning effect. This suggests that the subjects had no specific difficulty in learning to control the third hand with the foot and coordinating it with the two intrinsic hands.

The chronological order of the games was set from the one with least required motion to the one with highest amount of motion. However, the last game was reported as the easiest one for controlling the third hand by foot, the one with the most natural feeling and offering maximum sense of ownership towards the third hand.

Going through the responses to the questionnaire, another effect is also detectable: the influence of task type in the performance of the subjects and their feeling towards the control paradigm. Although participants get familiar with the setup during the first game, and the third game is supposed to be more difficult due to higher necessary amount of motion, they found the second game clearly more difficult compared to the first and the third games. This shows that apart from the experience, the task type also affects the approach of the subjects.

In the second game, the subjects were forced to move the foot-hand in opposite direction to the two hands. According to their comments, this makes the second game difficult, which corresponds to the relatively large time they need to carry out this task. On the other hand, the third game was more dynamic and more interesting which helps the participants in performing the task. Also in the first and the last games people were free to choose the sequence of their actions. Most of the participants could easily handle the multiple tasks of the third game. This hints at a large potential capacity of the human brain in multiple task handling. On the other hand, it was easiest for the participants to control the third hand by foot in the third game. They had more sense of ownership towards the third hand and they felt that it helped them more in mastering the simultaneous control of three hands. These results suggest that a combination of practice and appropriate tasks can enhance the process of learning to control multiple limbs simultaneously.

We note that the three proposed tasks were designed to investigate some basic questions about the control paradigm e.g. possibility of simultaneous control of three limbs, physical and mental burdens, but are no real surgical tasks. In addition, the experiment carried out involved a single session of three games, whereas it has been proven that the learning curve reaches a plateau after adequate number of sessions [38]. It would be interesting to study the learning curve of the subjects during multiple consecutive sessions for determining the total time required to reach the maximum possible level of expertise in controlling the third hand by foot. Also, subjects’ sense of ownership towards the third hand was assessed only through questionnaire. Other methods such as electrodermal activity could be used to assess the sense of ownership of the third hand in a more reliable and graded way.

Participants to our experiments were all high school or university engineering students, which may behave differently than surgical staff with different mean age and educational as well as professional background [39],[40]. In general, the present study suggests the feasibility of the controlling a third hand using a foot, which may be tested with specific tools and the target population of e.g. trained surgeons. The 2D tasks performed in virtual reality missed the third spatial dimension and the force feedback present in real life. Therefore, an experimental setup with these features might improve the embodiment and result in a better control of the third arm. Using the foot movements can be used to control more than two DoFs (taking its rotational degrees of freedom into account), and many tasks in fact involve few degrees of freedom. For instance typical laparoscopic surgery’s tools have only four DoFs.

## Conclusion

Although robotic arms have been commercialized for simple assistive surgical tasks such as holding the endoscope in laparoscopic surgery, they are generally controlled in a few discrete actions. In contrast, we studied the continuous control of a third arm, using foot movements. To our knowledge this is the first study on the simultaneous usage of the two hands and one foot for cooperative tasks. The results show that within a few minutes of practice the system’s users feel the control paradigm natural and easy. Only small physical and mental efforts were reported through the whole experiment, with an increasing sense of ownership towards the third hand. No specific order was detected in the movement of the limbs, indicating that the third hand controlled by the foot would come at a similar hierarchical level as the two hands. The level of difficulty of the task depended on the nature of the task as well as on the amount of practice received, and not directly on the amount of motion required during a game. These results suggest the possibility of commanding a robot by foot for collaborative actions with the hands with many kinds of applications, from surgeons operating with a robotic assistant to a worker using a robotic assistive device. In the tasks studied here subjects adapted fast to controlling three limbs and managed use them for performing tasks which required simultaneous motion. Coordination games such as these only hint at the vast possibility opened up by designing a proper interface and training strategy. This conclusion is also supported by the level of skill reached e.g. by organ players on the pedal keyboard.
